# Slime Mold Myosin Thick Filament Assembly Dissected

**DOI:** 10.1371/journal.pbio.0020392

**Published:** 2004-10-19

The movements needed to read this synopsis—turning the page, tracking along the lines, sitting, breathing—all require myosin, a molecular motor in muscle that transforms chemical energy into small but deliberate motions. But beyond these macro-movements, myosin is also required for the micro-movements of individual cells and their organelles and for determining cellular architecture.

There are many different myosins, but they all have the same general structure. At one end is a globular head, which is responsible for motor activity. This head binds ATP—the cell's power supply—and actin, an important component of the cytoskeleton of cells. Next comes a helical neck or lever region. Finally, there is a long helical tail, which has different and somewhat poorly understood functions in the different myosins.

Myosin II, the classical form of myosin found in essentially all eukaryotic cells, is constructed from two heavy chains (which contain the three regions described above) and two pairs of light chains (which stabilize the neck region). The long helical tail of myosin II is formed by the two heavy chains wrapping around each other and is involved in getting myosin II to the right place in the cell, as well as in assembling it into filaments.

Individual myosin II molecules can make tiny molecular motions. ATP cleavage induces a shape change in the globular head, which is transmitted to the lever region of the molecule. Angular rotation of this region moves the myosin along the actin filament. But to achieve the larger movements that are necessary to, for example, split cells apart during cell division, individual myosin II molecules group together to form highly regular bipolar structures called bipolar thick filaments (BTFs). In these, the globular myosin heads are positioned on either side of the filament, and the tail regions are clustered in the middle. This geometry enables myosin II molecules in thick filaments to pull from either side, generating contractile forces.

James Spudich's team has been studying the assembly of these thick filaments in the slime mold Dictyostelium discoideum, an organism beloved by developmental and cellular biologists because of its simple development and ease of manipulation. In the present study, the researchers examined the physical properties of various fragments of the myosin tail to find out how the self-assembly and disassembly of the BTFs are regulated. They already knew that the addition of phosphate groups on three specific threonine amino acid residues in this region (through a chemical reaction called phosphorylation) is important for regulating BTF assembly; they knew this from studies showing that mutation of these residues to aspartic acid, which mimics phosphorylated threonine, inhibits BTF formation. Here, the researchers show that a specific tail fragment of the myosin heavy chain containing the three crucial threonine residues assembles into a structure with some, but not all, of the properties of BTFs. However, replacing these threonine residues with aspartic acid prevents any self-assembly of the fragment.

Further experiments in which different tail regions were nibbled away and the assembly properties of the remaining fragments were determined suggest that the myosin tail contains a series of elements that correlate with the distribution of charged amino acids along the tail, some of which favor assembly and some of which favor disassembly. But it's not just the tail that is important. For myosin II to form fully fledged BTFs of a defined size, it seems that the addition of some kind of globular head—in these experiments one composed of green fluorescent protein so that it could be examined—is necessary. The overall result is a molecule that is finely poised to self-assemble into BTFs in response to one or two charge changes produced by phosphorylation. Consequently, the myosin contractile system can respond rapidly to environmental changes.

Although Dictyostelium myosin II is somewhat different from vertebrate myosin II, the general principle by which myosin assembly and disassembly are regulated seems likely to hold for other myosins and so might throw light onto human disorders that involve myosin defects. But more fundamentally, similar principles may hold for spatial and temporal regulation of the many other macromolecular assemblies that are at the heart of cell and developmental biology.

